# Changes in Nursing Students' Career Choices Following the COVID-19 Pandemic in China

**DOI:** 10.3389/fpsyt.2021.657021

**Published:** 2021-04-13

**Authors:** Wei Bai, Hai-Tao Xi, Qianqian Zhu, Zhiwen Wang, Lin Han, Pan Chen, Hong Cai, Yan-Jie Zhao, Li Chen, Zong-Mei Ge, Mengmeng Ji, Hongyan Zhang, Bing-Xiang Yang, Shuo Liu, Teris Cheung, Gabor S. Ungvari, Fengrong An, Yu-Tao Xiang

**Affiliations:** ^1^Unit of Psychiatry, Department of Public Health and Medicinal Administration, Institute of Translational Medicine, Faculty of Health Sciences, University of Macau, Macao, China; ^2^Centre for Cognitive and Brain Sciences, University of Macau, Macao, China; ^3^Institute of Advanced Studies in Humanities and Social Sciences, University of Macau, Macao, China; ^4^Department of Psychiatry, Jilin University Nursing College, Changchun, China; ^5^School of Nursing, Capital Medical University, Beijing, China; ^6^Beijing Key Laboratory of Mental Disorders Beijing Anding Hospital, The National Clinical Research Center for Mental Disorders, The Advanced Innovation Center for Human Brain Protection, School of Mental Health, Capital Medical University, Beijing, China; ^7^School of Nursing, Peking University, Beijing, China; ^8^School of Nursing, Lanzhou University, Lanzhou, China; ^9^School of Health Sciences, Wuhan University, Wuhan, China; ^10^School of Nursing, Hong Kong Polytechnic University, Hong Kong, China; ^11^Division of Psychiatry, School of Medicine, University of Western Australia/Graylands Hospital, Perth, WA, Australia; ^12^Department of Psychiatry, University of Notre Dame Australia, Fremantle, WA, Australia

**Keywords:** career choice, coronavirus disease 19, nursing students, pandemic, cross-sectional study

## Abstract

**Background:** Health professionals including nurses have experienced heavy workload and great physical and mental health challenges during the coronavirus disease 19 (COVID-19) pandemic, which may affect nursing students' career choices. This study examined the changes in nursing students' career choices after the onset of the COVID-19 pandemic in China.

**Methods:** This study was conducted in five University nursing schools in China between September 14, 2020 and October 7, 2020. Career choices before and after the COVID-19 pandemic were collected and analyzed.

**Results:** In total, 1,070 nursing students participated in the study. The reported choice of nursing as future career increased from 50.9% [95% confidence interval (CI): 47.9–53.9%] before the COVID-19 pandemic to 62.7% (95%CI: 59.8–65.6%) after the onset of COVID-19 pandemic. Students who chose nursing as their future career following the COVID-19 outbreak had less severe depression and anxiety compared to those who did not choose nursing, but the associations of depression and anxiety with career choice disappeared in multivariable analyses. Binary logistic regression analysis revealed that male gender [odds ratio (OR) = 0.68, 95% CI: 0.50–0.91], rural residence (OR = 1.53, 95%CI: 1.17–2.00), fourth year students (OR = 0.50, 95%CI: 0.35–0.72), negative experiences during the COVID-19 pandemic (OR = 0.66, 95%CI: 0.47–0.92), and good health (OR = 4.6, 95%CI: 1.78–11.87) were significantly associated with the choice of nursing as future career after the onset of the COVID-19 pandemic.

**Conclusions:** The COVID-19 pandemic appeared to have a positive influence on the career choice of nursing among Chinese nursing students.

## Introduction

The coronavirus disease (COVID-19) was first reported in China at the end of 2019, and was later declared as a public health emergency of international concern in late January 2020 and a worldwide pandemic on March 11, 2020 (1) by the World Health Organization (WHO) (2). By March 2020, the COVID-19 pandemic was well-controlled in some countries and territories, such as China (3).

Frontline health professionals, particularly nurses, played a key role in combating the COVID-19 pandemic (4). However, frequent exposure to COVID-19 cases, heavy workload and long working hours resulted in physical and mental burnout, emotional distress and psychiatric problems (5, 6) among healthcare workers including severe anxiety and depressive symptoms, and sleep disturbances (4, 7–10). An international study found that physical symptoms (i.e., headache and sore throat) were common in healthcare workers during the COVID-19 outbreak, which was associated with more severe anxiety, depression, stress, and post-traumatic stress disorder (11). Another study conducted in New York healthcare workers during the COVID-19 pandemic found that compared to attending physicians, nurses/advanced practice providers had more common mental health problems including acute stress, and depressive and anxiety symptoms (12). A meta-analysis revealed that the prevalence of anxiety, depressive, and insomnia symptoms in healthcare workers during the COVID-19 outbreak was 23.2, 22.8, and 38.9%, respectively (13). These consequences could affect nurses' quality of work and communication with patients and families, and even compromise workplace safety. A meta-analysis found that the overall prevalence of workplace violence was 62.4% in Chinese health professionals (14). Therefore, the cumulative stress placed on nursing staff may lead to job dissatisfaction and career changes during the COVID-19 pandemic. In Egypt, nurses in COVID-19 hospitals reported higher stress level (75.2%), heavier workload (98.6%), and lower satisfaction level (51.0%) compared to those working in the non-COVID-19 hospital (15). Only 4.8% of nurses in the COVID-19 hospital reported no intention to leave their job (15).

In a recent multicenter study involving 7,600 postdoctoral researchers worldwide regarding their work and career intentions (16), 61% of participants reported that the COVID-19 pandemic had negatively affected their career prospects. A study of 120 medical students in pediatric services (17) found no significant difference in students' career choices after the COVID-19 pandemic, but the proportion of choosing a medical career decreased from 72.6% to 68.3%, but 66.7% reported that the COVID-19 pandemic had strengthened their career choice to be pediatricians (17).

To date, no studies on the impact of the COVID-19 pandemic on nursing students' career choice have been published, which gave the impetus to examine the career choice of nursing students before and after after the onset of the COVID-19 pandemic, and explore the possible reasons for the change of career choice.

## Participants and Methods

### Participants and Study Settings

This was a multicenter, cross-sectional study conducted in five university nursing schools (Peking University, Capital Medical University, Jilin University, Lanzhou University, and Wuhan University) in China between September 14, 2020 and October 7, 2020. Participating universities are distributed in different regions of China, which enhanced the representativeness of the study sample. In order to avoid contagion, traditional face-to-face interviews were not adopted. Following other epidemiological studies (18, 19), the assessment was completed using the QuestionnaireStar application embedded with WeChat, which is a Smartphone-based social communication program with over 1 billion users in China. WeChat has been used in teaching activities in the participating universities, therefore all students are WeChat users. A Quick Response code (QR Code) linked to the assessment was distributed by WeChat among college students using the snowball sampling method. Inclusion criteria were: (1) undergraduate nursing students in universities, (2) aged between 15 and 28 years, and (3) able to understand Chinese and willing to provide electronical written informed consent. All nursing students in the participating nursing schools were consecutively invited to participate in the survey during the study period.

### Data Collection

A standard data form for this study was used to collect basic sociodemographic characteristics and information related to career choice. The following COVID-19 related questions were asked: whether they served as a volunteer in clinical settings during the COVID-19 pandemic; whether they had negative experiences (such as physical or verbal abuse) during the COVID-19 pandemic; whether they frequently used social media during the COVID-19 pandemic.

The following questions on career choice were asked: whether they chose nursing as their future career before and after the COVID-19 pandemic; whether they were interested in medicine before and after the COVID-19 pandemic. The Chinese version of the 2-item Patient Health Questionnaire (PHQ-2) was used to measure severity of depressive symptoms (20, 21). The total score of PHQ-2 ranges from 0 to 6, with a higher score indicating more severe depressive symptoms. The 7-item Generalized Anxiety Disorder scale (GAD-7)—Chinese version assessed the severity of anxiety symptoms with a higher score representing more severe anxiety symptoms (22, 23).

This study was approved centrally by the participating nursing schools and their ethics committees [Approval No.: (2020) Keyan (No. 10)]. Written informed consent to participate in this study was provided by the participants or legal guardians of those younger than 18 years.

### Statistical Analysis

All analyses were performed with SPSS, Version 24.0 (SPSS Inc., Chicago, Illinois, USA). Normal distribution of continuous variables was tested using P-P plots. Chi-square tests, independent two samples *t*-tests, and Mann–Whitney *U*-tests were conducted to compare socio-demographic and clinical variables between students who chose and those who did not choose nursing as future career after the COVID-19 pandemic. McNamar-test was used to compare nursing students' career choice before and after the COVID-19 pandemic. Binary logistic regression analysis was used to determine the independent correlates of career choice following the COVID-19 pandemic. Variables with significant differences in univariate analyses were included as independent variables, while career choice after COVID-19 pandemic was the dependent variable. Significance level was set at 0.05 (two-sided).

## Results

### Participant Characteristics

**Figure 1:**
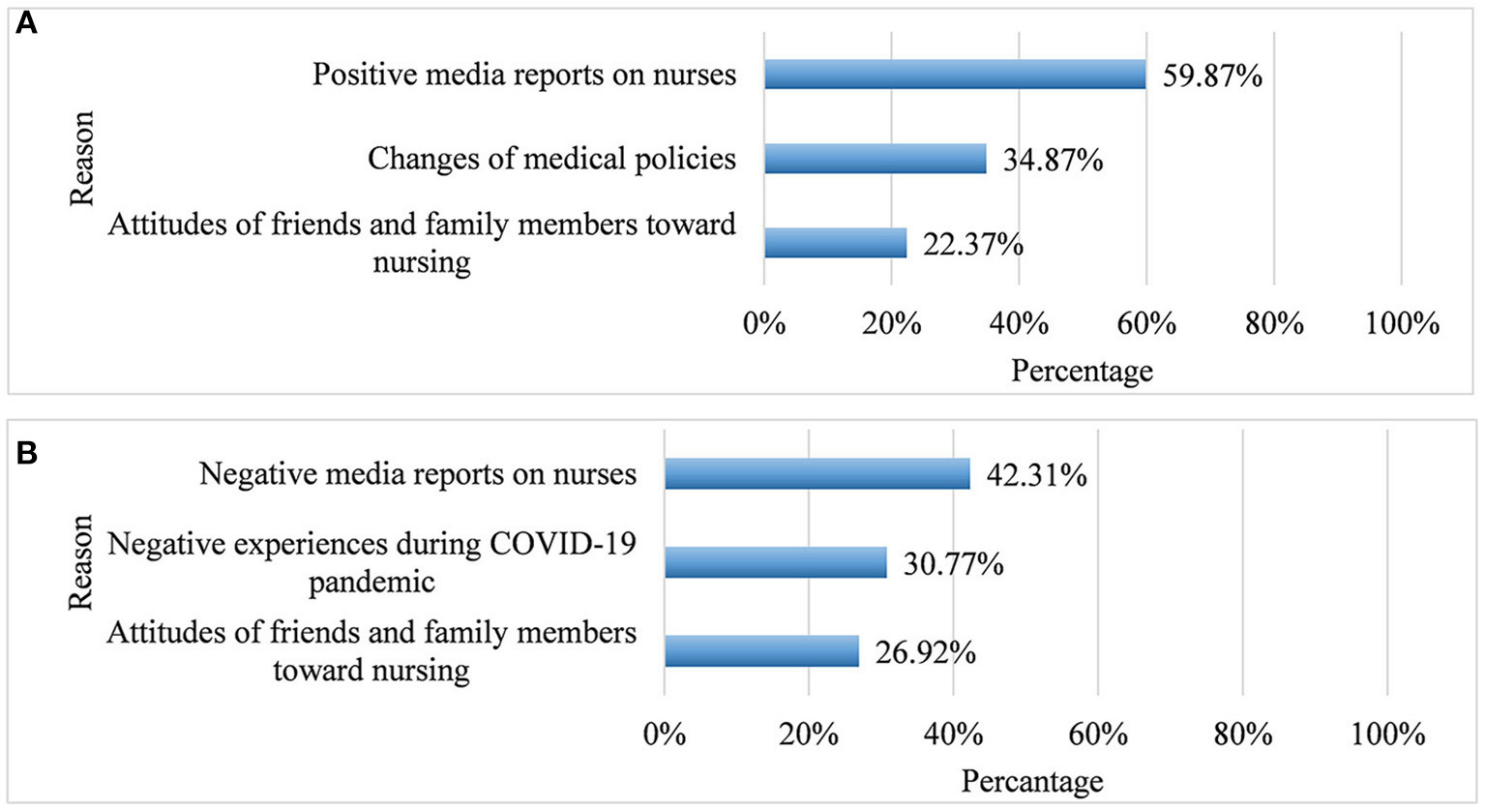
Causes of career choice change. **(A)** Career choice change from “not choosing nursing before COVID-19 pandemic” to “choosing nursing after COVID-19 pandemic” (*N* = 152). **(B)** Career choice change from “choosing nursing before COVID-19 pandemic” to “not choosing nursing after COVID-19 pandemic” (*N* = 26).

Of the 1,121 nursing students who were consecutively invited to participate in the survey, 1,070 met study entry criteria and completed the assessment, yielding a participation rate of 95.5%. About half of the students (50.9%; 95%CI: 47.9–53.9%) reported choosing nursing as their future career before the onset of the COVID-19 pandemic, while 62.7% (95%CI: 59.8–65.6%) chose nursing following the COVID-19 pandemic (OR = 5.85, 95% CI:4.05–8.44, *p* < 0.001). Table 1 shows the demographic and clinical characteristics and career choice of the sample after the COVID-19 pandemic. There were significant differences in terms of age, gender, residence, school grade, perceived health status, negative experiences during COVID-19 pandemic, being interested in medicine before COVID-19 pandemic, and choosing nursing as career before COVID-19 pandemic (all *p*-values < 0.05) between nursing students who chose and those who did not choose nursing as future career after COVID-19 pandemic. Students who did not choose nursing as their future career had more severe depressive (*p* = 0.005) and anxiety symptoms (*p* = 0.001) compared to those who chose nursing. Figure 1 summarizes the common reasons for career choice change in nursing students. The most common reason “for career change from” “not choosing nursing before the pandemic” to “choosing nursing after the onset of the COVID-19 pandemic” was the positive media reports on nurses, while the most common reason for change in the opposite direction was negative media reports and adverse experiences during the pandemic.

**Table 1:** Sociodemographic and clinical characteristics of the participants.

**Variable**	**Total (*N* = 1,070)**		**Choosing nursing as career after the onset of the COVID-19 pandemic**						
			**No (*n* = 399)**		**Yes (*n* = 671)**		**Statistics**		
	***N***	**%**	***N***	**%**	***N***	**%**	**χ^2^**	**df**	P^*^
Male gender	265	24.8	117	29.3	148	22.1	7.09	1	**0.008**
Only child	457	42.7	179	44.9	278	41.4	1.20	1	0.273
Rural residence	457	42.7	152	38.1	305	45.5	5.54	1	**0.019**
School grade							23.22	3	**<0.001**
First year	287	26.8	84	21.1	203	30.3			
Second year	237	22.1	80	20.0	157	23.4			
Third year	249	23.3	93	23.3	156	23.2			
Fourth year	297	27.8	142	35.6	155	23.1			
Working as volunteers during COVID-19 pandemic	231	21.6	94	23.6	137	20.4	1.45	1	0.227
Negative experiences during COVID-19 pandemic	188	17.6	91	22.8	97	14.5	12.05	1	**0.001**
Frequent use of social media during COVID-19 pandemic	778	72.7	280	70.2	498	74.2	2.06	1	0.151
Perceived economic loss during COVID-19 pandemic							3.15	2	0.207
No or mild	444	41.5	176	44.1	268	39.9			
Moderate	557	52.1	194	48.6	363	54.1			
Severe	69	6.4	29	7.3	40	6.0			
Perceived economic status							2.63	2	0.268
Poor	218	20.4	89	22.3	129	19.2			
Fair	776	72.5	278	69.7	498	74.2			
Good	76	7.1	32	8.0	44	6.6			
Perceived health status							28.36	2	**<0.001**
Poor	23	2.1	16	4.0	7	1.0			
Fair	449	42.0	197	49.4	252	37.6			
Good	598	55.9	186	46.6	412	61.4			
Being interested in medicine before COVID-19 pandemic							119.63	2	**<0.001**
No or a little	151	14.1	104	26.1	47	7.0			
Fair	512	47.9	213	53.4	299	44.6			
Very much	407	38.0	82	20.6	325	48.4			
Choosing nursing as future career before COVID-19 pandemic	545	50.9	26	6.5	519	77.3	502.31	1	**<0.001**
	**Mean**	**SD**	**Mean**	**SD**	**Mean**	**SD**	***t/Z***	**df**	***P***
Age (years)	19.7	1.4	19.9	1.4	19.6	1.4	2.6	1.068	**0.010**
PHQ-2 total	1.03	1.24	1.19	1.35	0.94	1.16	2.84	-^†^	**0.005**
GAD-7 total	3.14	3.92	3.76	4.45	2.77	3.53	3.31	-^†^	**0.001**

† *Mann-Whitney U-test*.

* *Bold values: P < 0.05*.

*COVID-19, Coronavirus Disease 2019; df, degree of freedom; GAD-7, 7-item Generalized Anxiety Disorder Scale; PHQ-2, the 2-item Patient Health Questionnaire; SD, standard deviation*.

### Independent Correlates of Career Choice

Binary logistic regression analysis revealed that nursing students, who lived in rural areas, had perceived good health status, and chose nursing as future career before the COVID-19 pandemic, were more likely to choose nursing as future career after the onset of the COVID-19 pandemic. Compared to the first-year students, those in second, third, and fourth years were less likely to choose nursing as future career after the onset of the COVID-19 pandemic (all *p*-values < 0.05; Table 2).

**Table 2:** Independent correlates of choosing nursing as future career after the COVID-19 pandemic (binary logistic regression analyses).

**Variables**	**Binary logistic regression analyses**^†^					
	P^*^	**OR**	**95% CI**	*P*^‡^^*^	**OR**	**95% CI**
Male gender	0.140	0.74	0.49–1.11	**0.009**	0.68	0.50–0.91
Rural residence	**0.001**	1.87	1.29–2.72	**0.002**	1.53	1.17–2.00
School grade						
First year	**-**	1	**-**	-	1	-
Second year	**0.002**	0.44	0.27–0.74	0.519	0.88	0.60–1.29
Third year	**<0.001**	0.31	0.18–0.52	0.187	0.77	0.53–1.13
Fourth year	**<0.001**	0.20	0.12–0.33	**<0.001**	0.50	0.35–0.72
Negative experiences during COVID-19 pandemic	0.942	1.02	0.65–1.60	**0.013**	0.66	0.47–0.92
Perceived health status						
Poor	**-**	1	**-**	-	1	-
Fair	0.064	3.47	0.93–12.97	**0.026**	2.91	1.13–7.45
Good	**0.015**	5.24	1.38–19.83	**0.002**	4.60	1.78–11.87
PHQ-2 total	0.333	1.10	0.90–1.35	0.901	0.99	0.86–1.45
GAD-7 total	0.146	0.95	0.90–1.02	0.162	0.97	0.92–1.01
Choosing nursing as future career before COVID-19 pandemic	**<0.001**	66.90	44.45-108.01	**—**	**—**	**—**

† *There was collinearity between age and grade, and between being interested in medicine before COVID-19 pandemic and choosing nursing as career before COVID-19 pandemic, therefore age and being interested in medicine before COVID-19 pandemic were not entered as independent variables*.

‡ *Without “Choosing nursing as career before COVID-19 pandemic” as an independent variable*.

* *Bold values: P < 0.05*.

*CI, confidential interval; COVID-19, Coronavirus Disease 2019; GAD-7, 7-item Generalized Anxiety Disorder Scale; OR, odds ratio; PHQ-2, the 2-item Patient Health Questionnaire*.

To clarify the contributions of other variables independent of choosing nursing as future career before COVID-19 pandemic, the binary logistic regression analysis was repeated after removing “choosing nursing as future career before COVID-19 pandemic.” The results show that nursing students who lived in rural areas and had perceived fair and good health status were more likely to choose nursing as future career after the COVID-19 pandemic. In addition, male students, fourth-year students and students who had negative experiences during the COVID-19 pandemic were less more likely to choose nursing as future career after the COVID-19 pandemic (all *p*-values < 0.05; Table 2).

## Discussion

To the best of our knowledge, this was the first study that compared nursing students' career choice before and after the onset of the COVID-19 pandemic. Prior to the COVID-19 pandemic, 50.9% of Chinese nursing students reported choosing nursing as their future career, and the corresponding figure increased to 62.7% after the pandemic, both of which are higher than the previous finding in a study from Taiwan (34.6%) (24), but considerably lower than the corresponding figures from the USA (99.4%) and Turkey (81.1%) (25). Apart from the confounding effects owing to different demographic characteristics between studies, lower proportion of nursing students who chose nursing as their future career in this study compared to their Western counterparts could be due to the following reasons. First, workplace violence is common in clinical settings in China (14). Second, due to insufficient health resources in China, the nurse/patient ratio is low, resulting in heavy workload and long working hours (26, 27). Third, due to historical reasons, nurses have a relatively lower wages (28) and social status than other occupations, such as doctors, police officers and teachers (29). The increased proportion of nursing students choosing nursing as their future career after the COVID-19 pandemic could be associated with a number of reasons. Nurses who volunteered working at frontline hospitals during the pandemic were widely applauded by the public media. This conveyed a positive image of nurses and may have encouraged some nursing students to choose nursing as their future career. In addition, policies have been implemented to improve nurses' welfare following the COVID-19 pandemic (30–32).

Gender, residence, school grade, perceived health status, career choice before pandemic, and negative experiences during the COVID-19 pandemic, were associated with choosing nursing as future career after the COVID-19 pandemic. Compared with female students, male nursing students were less likely to choose nursing as their future career after the COVID-19 pandemic (OR = 0.68, 95% CI: 0.50–0.91), which is consistent with the data that nurses are predominantly females in most countries (33). In traditional Chinese societies, men are usually viewed as the “pillar” of families and are expected to have a higher income than women. Consequently, some male students did not choose nursing as their future career due to lower wages and social status in China. In this study, nursing students living in rural areas were more likely to choose nursing as their future career than their counterparts living in urban areas, which may be related to the poor economic status and fewer employment opportunities in rural areas of China. In contrast, due to the wider social network and connections, nursing students in urban areas usually have more job choices than those living in rural areas and may not choose nursing as their future career. This is similar to previous findings of a multicenter study that medical students brought up and educated in rural areas were less likely to work in rural areas (34).

Both depressive and anxiety symptoms were less severe in students who chose nursing as future career after the COVID-19 pandemic than students who did not choose nursing. Increased fear of COVID-19 was associated with higher likelihood of psychological distress and professional turnover intentions in frontline nurses (35). We hypothesize that nursing students who experienced depressive and anxiety symptoms or psychological distress due to fear of COVID-19 may have been influenced by such experiences in their career choice. However, the association of depressive and anxiety symptoms with career choice disappeared in multivariate analysis, probably because the association between mental health and career choice was moderated by other variables. In binary logistic regression analysis, students who had negative experiences during the COVID-19 pandemic were less likely to choose nursing as future career, while those who had good health status were more likely to choose nursing as future career after the COVID-19 pandemic. This could be explained by the following reasons. On one hand, negative experiences and relevant appraisals, such as fear of infection, uncertainty, stigmatization, discrimination from neighbors, unsafe workplace and risk of infection, are possible risk factors for psychological distress, particularly post-traumatic stress reactions, in healthcare workers (36, 37), which could have lowered the likelihood of choosing nursing as a future career. On the other hand, good personal health status and working in well-organized medical units, where protected environment is provided (38, 39) and precautionary measures are regularly taken (40), together with support from family, friends, supervisors, colleagues and society, have been proven to be important factors building up resilience against the development of distress (38–41), thereby increasing the likelihood of choosing nursing as future career.

Multivariate analysis also revealed that compared to first year students, those in senior grades were less likely to choose nursing as future career after the COVID-19 pandemic. Interestingly, the likelihood decreased with school grade: ORs = 0.44 (95% CIs: 0.27–0.74) in second year, ORs = 0.31 (95% CIs: 0.18–0.52) in third year, and ORs = 0.20 (95% CIs: 0.12–0.33) in fourth-year students. After removing the independent variable “choosing nursing as career before COVID-19 pandemic” which contributed the most to the model (OR = 66.9), fourth year students were still less likely to choose nursing as future career compared to the first grade students (OR = 0.50, 95% CI: 0.35–0.72), which is consistent with the findings in medical students (42). A recent study of Chinese medical students found that senior grade students were less likely to be volunteers combating the COVID-19 pandemic compared to junior and middle grade students (42), probably due to the risk of COVID-19 infection during the pandemic and relatively low income for junior doctors. Intention to be volunteers and career choice in medical or nursing students may be associated with workplace safety such as provision of personal protective equipment (43, 44). If the workplace is unsafe, many students may be unwilling to be volunteers, doctors or nurses. Therefore, we assume that higher grade nursing students were more aware of the potential risks and increased workload in clinical settings during the pandemic, therefore they were less likely to choose nursing as future career. Nursing students in good health status were more likely to choose nursing as future career, probably because these students felt they had the capacity to handle heavy clinical workload.

In this study, significantly more nursing students chose nursing as their future career after the pandemic than before the pandemic (*p* < 0.001). Of the 152 nursing students who changed their intention from “not choosing nursing before the pandemic” to “choosing nursing after the pandemic,” more than half mentioned that the reason was due to positive media reports on nurses. In China, many relevant policies have been released (30–32) to improve frontline health professionals' welfare during and after the COVID-19 pandemic, which probably increased the appeal of a nursing career. Of the 26 nursing students who changed their intention from “choosing nursing before the pandemic” to “not choosing nursing after the pandemic,” the most common reason was negative media reports and experiences. It is likely that negative factors as high risk of infection, heavy clinical workload (45), discrimination (46), sexual harassment (47) and workplace violence, discouraged nursing students to choose nursing career.

The strengths of this study are its large sample size, the representativeness of the study sample, and the comparisons of career choice before and after the onset of the COVID-19 pandemic. However, several limitations should be noted. First, due to the cross-sectional design, the causal relationships between career choice and other variables could not be determined. Second, the data on career choice prior to the COVID-19 pandemic were retrospectively collected, therefore, the possibility of recall bias could not be excluded. Third, a few factors relevant to career choice, such as social support, family members' career and students' negative experiences during the pandemic, were not recorded.

In conclusion, the COVID-19 pandemic appeared to have a positive influence on the choice of nursing as future career among Chinese nursing students. To increase the nursing workforce, effective measures should be implemented to promote nursing as a suitable career choice for nursing students.

## Data Availability Statement

The Clinical Research Ethics Committee of Beijing Anding Hospital at Capital Medical University that centrally approved the study prohibits the authors from making the research data set publicly available. Readers and all interested researchers may contact Dr. Feng-Rong An (Email address: afrylm@sina.com) for details. Dr. An could apply to the Clinical Research Ethics Committee of Beijing Anding Hospital for the release of the data.

## Ethics Statement

The studies involving human participants were reviewed and approved by the participating nursing schools (Peking University, Capital Medical University, Jilin University, Lanzhou University, and Wuhan University) and their Ethics Committees [Approval No.: (2020) Keyan (No. 10)]. Written informed consent to participate in this study was provided by the participants' legal guardian/next of kin.

## Author Contributions

WB, H-TX, QZ, ZW, LH, and PC completed the data collection, analysis, interpretation, and drafted the manuscript. HC, Y-JZ, LC, Z-MG, MJ, HZ, B-XY, and SL completed the data collection, analysis, and interpretation. TC and GU finished the critical revision of the manuscript. FA and Y-TX completed study design, the data collection, analysis, interpretation, and drafted the manuscript. All the authors finished the approval of the final version for publication.

## Conflict of Interest

The authors declare that the research was conducted in the absence of any commercial or financial relationships that could be construed as a potential conflict of interest.
